# The Long Path from QTL to Gene

**DOI:** 10.1371/journal.pgen.1002975

**Published:** 2012-09-20

**Authors:** Norman R. Drinkwater, Michael N. Gould

**Affiliations:** McArdle Laboratory for Cancer Research, Department of Oncology, University of Wisconsin School of Medicine and Public Health, University of Wisconsin-Madison, Madison, Wisconsin, United States of America; Cincinnati Children's Hospital Medical Center, United States of America

The construction in the 1990s of high density genetic maps of the mouse and rat based on simple sequence length polymorphisms led to an explosion of activity directed toward the identification of quantitative trait loci (QTLs) that control a broad array of normal and abnormal biology. More than 3,900 mouse and nearly 1,000 rat QTLs have been mapped by linkage analysis in studies of, among others, behavior, bone morphogenesis, cardiovascular function, and metabolism, as well as diseases including arthritis, diabetes, and cancer [1], [2].

The development of cancer is a complex, multi-step process that begins with a genetic or epigenetic event in a normal cell (initiation), followed by expansion and evolution of the initiated cells during the promotion stage, and culminating with the acquisition of malignant phenotypes, including invasiveness and metastatic potential, during tumor progression [3]. That complexity has prompted many investigators, including Hunter and colleagues (whose work is presented in this issue, [4]), to pursue the identification of cancer modifier genes, QTLs that alter cancer development in rodents. Compelling motivations for this work include the expectation that the genes underlying the QTLs will provide paradigms for understanding genetic variation in human cancer risk, that identification of the relevant genes will yield insights into pathways critical for carcinogenesis, and that these genes and the pathways they represent will provide novel targets for intervention to prevent or treat cancer. Nearly 250 QTLs that modify cancer risk or pathogenesis have been mapped in mice or rats [1], [2], [5]. However, despite this wealth of genetic information, only a small handful of these QTLs have been identified at the molecular level, as specific genes or non-coding elements. The paucity of molecular identifications applies more broadly, and Flint et al. estimated that, by 2005, less than 1% of rodent QTLs had been carried to the level of the gene [6].

**Figure 1:**
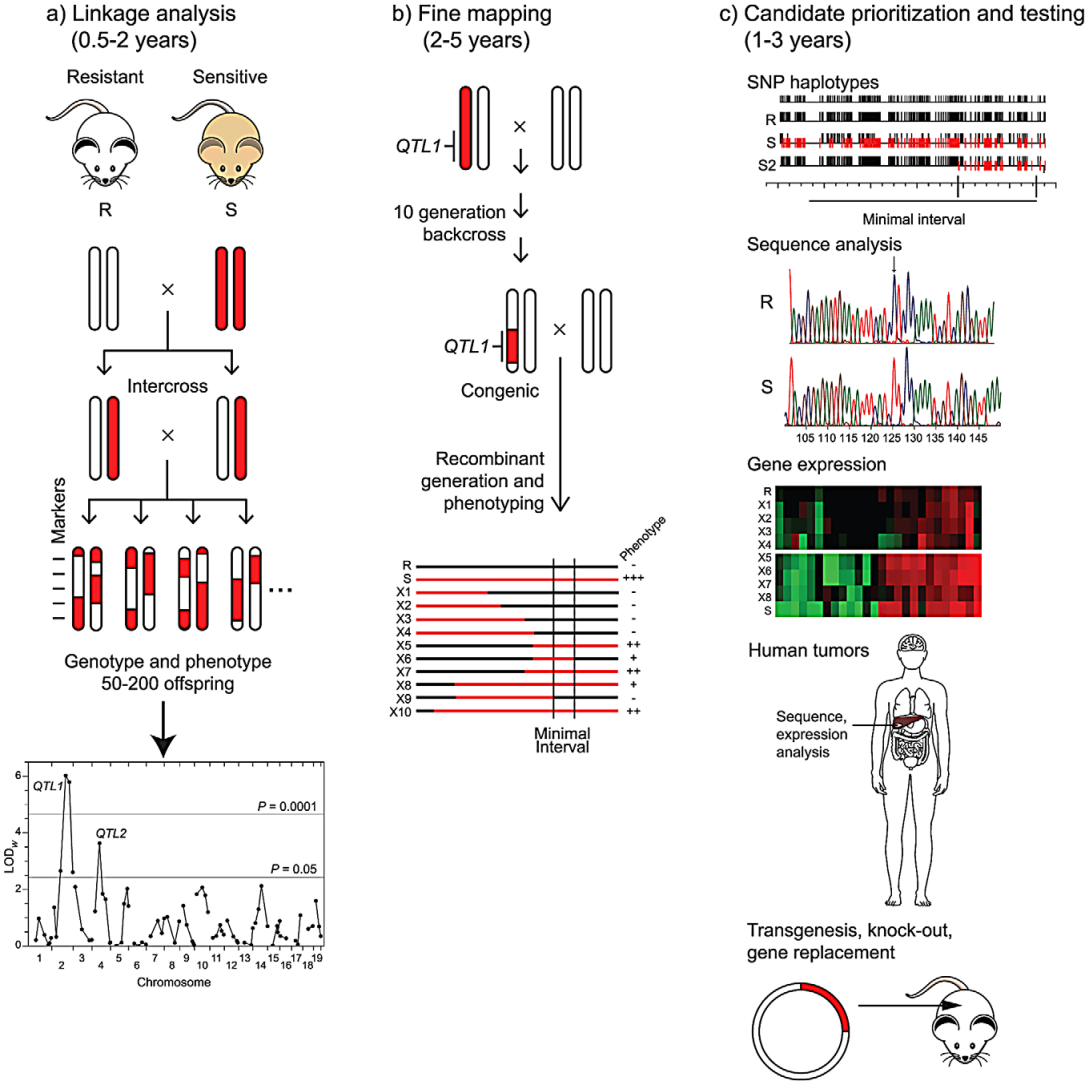
From QTL to causative gene. Three phases of QTL gene identification are depicted, with the length of time for each phase shown for phenotypes, such as cancer endpoints, that may take several months to a year to assess. a) Linkage analysis. Two inbred mouse or rat strains that differ in cancer risk are intercrossed. Chromosomal segments from the resistant strain (R) are shown in white and those from the susceptible strain (S) are shown in red. After phenotyping 50–200 progeny and genotyping them at approximately 100 markers, linkage analysis reveals two QTLs (*QTL1* and *QTL2*) on chromosomes 2 and 4. b) Fine mapping. A congenic line carrying the *QTL1* chromosomal region from the sensitive strain on the genetic background of the resistant strain is constructed by repeated backcrossing to the resistant strain, with selection for markers from the sensitive strain in the desired interval. The heterozygous congenic is backcrossed to the resistant strain to produce recombinant lines (X1 to X10) that carry various segments of the region from the sensitive strain (shown as red lines). A smaller chromosomal region (Minimal Interval) containing *QTL1* is inferred from phenotypic analysis of the lines. c) Candidate prioritization and testing. Candidate genes within the minimal interval may be prioritized by several complementary approaches. Comparison of SNP haplotypes for the resistant and sensitive strains (R and S) to those for other strains, such as S2, another sensitive strain with a QTL mapping to the same region as for S, may allow definition of a smaller interval for *QTL1*. Direct sequence analysis of alleles in the resistant (R) and sensitive (S) strains may identify polymorphisms with potential biological consequences. Differential expression of candidate genes in tumors or appropriate normal tissues from sensitive and resistant recombinant lines may be assessed by transcriptome analysis. Mutations or altered expression of the orthologue of a candidate may be observed in human tumors. Ultimately, a candidate gene may be tested directly for its role in cancer development by modifying the genome of the resistant or sensitive strain through transgenesis or homologous recombination.

The slow accrual of gene identifications is a consequence of the long path from QTL to gene (Figure 1). The starting point for most cancer QTL studies is the observation of significant variation in cancer risk among inbred strains. For virtually any tissue site, large (up to 100-fold) differences in the incidence or multiplicity of spontaneous or induced tumors may be found in the literature going back to the development of inbred strains early in the last century. Linkage analysis of segregating backcrosses or intercrosses between a pair of inbred strains with divergent cancer phenotypes may lead to the identification of one or more QTLs that control, for example, tumor incidence, multiplicity, latency [7], [8], or, as in the work by Hunter and colleagues (Faraji et al., this issue [4]), metastatic potential. Other experimental designs, including crosses between congenic or chromosome substitution strains, may be used to increase the power to detect QTLs. An intrinsic limitation of this approach is that, owing to the quantitative, variable phenotype, the precision for mapping QTLs is typically low; even with large crosses and a high density of genetic markers, the resulting 1.5 LOD support interval may be 20 cM (around 40 Mb) and contain hundreds of genes. Mapping QTLs to higher resolution requires the time and resources to produce congenic lines that carry a limited interval of the high (or low) risk donor strain's genome on the genetic background of the other strain, followed by phenotypic analysis of recombinant lines derived from that congenic. This fine mapping may yield intervals of the order of one to a few megabases, with one to 40 potential candidate genes. A caveat to this approach is that genetic complexity, with multiple sub-intervals contributing to the phenotype, has been observed for cancer QTLs more often than not, expanding the hunt for the causative genes. Prioritization of candidates within the interval may be based on sequence analysis, taking advantage of the high density SNP maps available for a large number of strains or the recent whole-genome assemblies available for a handful of strains [9], [10]. Depending on knowledge of the site of action of the QTL (e.g., whether it is cell-autonomous or acts indirectly), gene expression analysis by microarray may also be used to prioritize candidates. The “gold standard” for proof that a particular candidate is the causative gene by transgenesis or allelic substitution by homologous recombination has been achieved in only a few cases, but analysis of gene knockout strains or demonstration of specific genetic or epigenetic alterations in the orthologue in human tumors has more often provided a weight of evidence in favor of a particular candidate.

The fact that metastases account for most cancer-related deaths led Hunter and colleagues to pursue QTLs that controlled the risk for metastasis in a transgenic, Polyoma-middle T (PyMT) model for breast cancer in mice, largely following the path depicted in Figure 1. More than a decade ago, they demonstrated the presence of a metastasis susceptibility gene on chromosome 9 in crosses between NZB and PyMT-FVB mice [7] and validated the existence of this modifier in chromosome substitution strains [8]. Faraji et al. [4] now describe the development and analysis of congenic mouse lines carrying various segments of proximal chromosome 9 from NZB mice on an FVB genetic background, allowing them to narrow the interval for the susceptibility QTL to a 21 megabase region. They used haplotype, DNA sequence, and gene expression data to prioritize the list of candidates and describe biological studies of one of them, *Cadm1*, in the present paper. *Cadm1*, also known as *Tslc1* (*Tumor suppressor in lung cancer 1*), is an immunoglobulin superfamily cell adhesion molecule. Based on their studies, the authors hypothesize that *Cadm1* expression suppresses metastasis by sensitizing tumor cells to elimination via immune surveillance. Their studies also demonstrate the complexities of identifying QTL genes. Despite the over-expression of *Cadm1* in NZB relative to FVB mice and the fact that the NZB chromosome 9 interval enhances metastasis, they found that ectopic expression of this candidate gene suppresses metastasis and that high expression of *Cadm1* in tumors is associated with improved survival in women with breast cancer. Thus, one or more additional modifier genes within the interval likely lead to the phenotype of enhanced metastasis in the congenic mice and remain unidentified.

The work by Hunter and colleagues represents a substantial investment in time and financial resources, much of which involves mouse breeding and maintenance. The current economic climate makes the launching of projects using similar approaches difficult. Fortunately, over the decade since the inception of the project, new rodent resources and methods have been developed making future analysis of complex traits in rodents more efficient in both time and resources.

The Faraji et al. [4] study began with extensive mapping of loci associated with susceptibility to metastasis, which took many years. In the near future, similar work could be done in a single series of phenotyping experiments using new mouse resources such as the Collaborative Cross (CC) [11] or the Diversity Outbred (DO) populations of mice [12]. In addition, once a QTL is fine-mapped, finding the causative genetic element will be facilitated by open access to mouse/rat whole genome sequences for multiple strains [10]. Likewise, validation of candidate genes will no longer require time-consuming production of knockout (KO) mice from engineered embryonic stem cells but can be obtained from various repositories generated from the mouse KOMP project [13], [14]. While the KOMP resources provide a wide variety of mutants, most are on a C57BL/6 background. Often phenotypes must be evaluated on other genetic backgrounds, which requires six to ten generations of backcrossing over a period of one or more years. Alternative approaches are now available for mice and rats that use Zn finger nucleases or TALENS to knockout or replace genes [15]. These technologies are able to produce mouse or rat KO founders in less than 3 months on almost any genetic background.

The future for the use of rodent models to unravel the genetic complexity of common disease is expanding due to the intriguing published studies in this area, such as that by Hunter and colleagues, coupled with emerging new powerful genomic technologies and animal resources.
